# Myocardial Ischemia Related to Common Cancer Therapy—Prevention Insights

**DOI:** 10.3390/life12071034

**Published:** 2022-07-12

**Authors:** Minerva Codruta Badescu, Oana Viola Badulescu, Dragos Viorel Scripcariu, Lăcrămioara Ionela Butnariu, Iris Bararu-Bojan, Diana Popescu, Manuela Ciocoiu, Eusebiu Vlad Gorduza, Irina Iuliana Costache, Elena Rezus, Ciprian Rezus

**Affiliations:** 1 Department of Internal Medicine, “Grigore T. Popa” University of Medicine and Pharmacy, 16 University Street, 700115 Iasi, Romania; minerva.badescu@umfiasi.ro (M.C.B.); popescu.diana@umfiasi.ro (D.P.); irina.costache@umfiasi.ro (I.I.C.); ciprian.rezus@umfiasi.ro (C.R.); 2 III Internal Medicine Clinic, “St. Spiridon” County Emergency Clinical Hospital, 1 Independence Boulevard, 700111 Iasi, Romania; 3 Department of Pathophysiology, “Grigore T. Popa” University of Medicine and Pharmacy, 16 University Street, 700115 Iasi, Romania; iris.bararu@umfiasi.ro (I.B.-B.); manuela.ciocoiu@umfiasi.ro (M.C.); 4 Hematology Clinic, “St. Spiridon” County Emergency Clinical Hospital, 1 Independence Boulevard, 700111 Iasi, Romania; 5 Surgery Department, “Grigore T. Popa” University of Medicine and Pharmacy, 16 University Street, 700115 Iasi, Romania; 6 1st Surgical Oncology Unit, Regional Institute of Oncology, 2-4 General Henri Mathias Berthelot Street, 700483 Iasi, Romania; 7 Department of Mother and Child Medicine, “Grigore T. Popa” University of Medicine and Pharmacy, 700115 Iasi, Romania; eusebiu.gorduza@umfiasi.ro; 8 Cardiology Clinic, “St. Spiridon” County Emergency Clinical Hospital, 700111 Iasi, Romania; 9 Department of Rheumatology and Physiotherapy, “Grigore T. Popa” University of Medicine and Pharmacy, 16 University Street, 700115 Iasi, Romania; elena.rezus@umfiasi.ro; 10 I Rheumatology Clinic, Clinical Rehabilitation Hospital, 14 Pantelimon Halipa Street, 700661 Iasi, Romania

**Keywords:** accelerated atherosclerosis, coronary spasm, coronary thrombosis, endothelial dysfunction, cancer, prevention, radiotherapy, chemotherapy

## Abstract

Modern antineoplastic therapy improves survival and quality of life in cancer patients, but its indisputable benefits are accompanied by multiple and major side effects, such as cardiovascular ones. Endothelial dysfunction, arterial spasm, intravascular thrombosis, and accelerated atherosclerosis affect the coronary arteries, leading to acute and chronic coronary syndromes that negatively interfere with the oncologic treatment. The cardiac toxicity of antineoplastic agents may be mitigated by using adequate prophylactic measures. In the absence of dedicated guidelines, our work provides the most comprehensive, systematized, structured, and up-to-date analyses of the available literature focusing on measures aiming to protect the coronary arteries from the toxicity of cancer therapy. Our work facilitates the implementation of these measures in daily practice. The ultimate goal is to offer clinicians the necessary data for a personalized therapeutic approach for cancer patients receiving evidence-based oncology treatments with potential cardiovascular toxicity.

## 1. Introduction

Early diagnosis and modern therapies have significantly improved the survival and quality of life in cancer patients. The 5-year relative survival rate for all cancer sites combined has increased by 19% over the past 30 years in the USA [1], and its steady increase over the past 20 years was found in all European regions as well [2]. Still, cancer therapy has harmful effects on the cardiovascular system, which can diminish the benefits obtained. Cardiovascular disease increases morbidity and mortality not only by itself but also by limiting the use of anticancer therapies [3], and to date, it is the main cause of mortality in cancer survivors [4]. 

Cancer treatment is based on three major pillars, namely surgery, chemotherapy (CT), and radiation therapy (RT), that can be used in various combinations, reflecting the great complexity of modern oncologic therapy. Cardiovascular complications of cancer therapy are multiple and may affect all heart structures as well as blood vessels. The cardiovascular toxicities of antineoplastic agents manifest as myocardial dysfunction and heart failure, coronary artery disease (CAD), valvular disease, arrhythmias, arterial hypertension, peripheral vascular disease and stroke, thromboembolic disease, pulmonary hypertension, and pericardial complications [5]. RT induces endothelial dysfunction and fibrosis. All components of the heart can be affected, which leads to CAD, cardiomyopathy, valvulopathy, arrhythmias, and pericardial disease [6]. 

Cancer treatments act unfavorably on coronary arteries. The main mechanisms are endothelial dysfunction, vasospasm, thrombosis, and fibrosis. The process of atheroma plaque formation is more intense and takes place at a much faster rate. Toxic effects of the cancer treatments can destabilize a normal endothelium or a pre-existing atheroma plaque. Therefore, patients can develop acute coronary syndromes (ACSs) or accelerated atherosclerosis and early CAD while on oncologic treatment. This risk is enhanced by the presence of classical cardiovascular risk factors and varies with the type, dose, and duration of cancer therapy [7]. In patients receiving both CT and RT, the risk of CAD is the highest: two or even three times higher than in patients receiving only RT [8,9]. 

The scope of our review is to realize a comprehensive, systematized, structured, and up-to-date analyze of the available literature focusing on measures aiming to protect the coronary arteries from the toxicity of cancer therapy. Cardiac toxicity exhibited by anticancer drugs is based on multiple mechanisms; therefore, extensive search on the *Web of Science* database was performed in order to collect the necessary data. Starting from the pathophysiological mechanisms of coronary artery dysfunction induced by cancer treatment, we provided reliable data regarding modern approaches to prevention. The 2016 European Society of Cardiology position paper on cancer treatments and cardiovascular toxicity mainly focuses on RT and three categories of anticancer drugs, namely antimetabolites, platinum compounds, and vascular endothelial growth factor pathway inhibitors, as the most important toxic agents for the coronary arteries [5]. Our work is concordant with this guideline.

## 2. Antimetabolites (5-FU, Capecitabine, Gemcitabine)

Fluoropyrimidines (FP), namely 5-fluorouracil (5-FU) and its oral prodrug capecitabine, are commonly used for the treatment of various solid tumors, e.g., breast, gastric, colorectal, and head and neck cancers, due to their wide antitumor effect and their synergic activity with other anticancer drugs [10]. Despite the benefits, cardiac adverse effects are very common. Only the cardiac toxicity of anthracyclines exceeds that of FP [11]. The reported incidence of cardiovascular toxicity ranges from 1% to 19% [12] and mainly manifests as CAD. Myocardial ischemia usually has clinical expression, but the existence of asymptomatic forms leads to an underestimation of the prevalence of 5-FU-induced CAD [13,14]. Gemcitabine is preferred in old or fragile patients due to its lower toxicity profile compared to other anticancer drugs. Meta-analyzes reported an overall incidence of cardiovascular adverse effects of 1% and myocardial infarction (MI) of 0.5% [15]. 

The mechanism of FP-related cardiovascular toxicity is very complex and not fully elucidated despite the long-term use of these compounds. The coronary artery spasm in the first place and thrombosis in the second place were identified as major pathogenic substrates [16,17]. Vasoconstriction is triggered by two mechanisms, and both are the consequence of vascular endothelial cell injury. The first is endothelium-independent and a result of the activation of the protein kinase C pathway [18]. The second is endothelium-dependent and mediated by high levels of endothelin-1 [19]. Transient coronary vasospasm may cause an episode of chest pain or unstable angina pectoris, whereas persistent vasospasm may produce an acute MI. This mechanism is considered responsible for the onset of acute MI during intravenous administration of 5-FU. Moreover, coronary artery vasospasm can be directly visualized during coronary angiography [11]. Interestingly, the multivessel coronary spasm is uncommon although the distribution of the drug is systemic. Epicardial vasospasm was typically observed in a single vessel during coronary angiography, usually the vessel supplying the largest territory of the myocardium. Of note, the bolus therapy of 5-FU was associated with a much lower risk of cardiac toxicity (5%) than continuous infusion (10–18%) because the half-life of the drug is 15–20 min, so it is eliminated quickly when given as a bolus [11]. 

The coronary spasm usually occurs in the absence of classic cardiovascular risk factors and significant coronary stenosis on angiography [20]. Moreover, the angiography is typically normal without evidence of a thrombotic event [21]. However, microthrombotic occlusions have been identified by scanning electron microscopy in animal studies [22]. 5-FU produces endothelial dysfunction, endothelial cells cytolysis, and denudation of the underlying internal elastic lamina, followed by activation of platelets and coagulation cascade and thrombus formation [23]. Accelerated thrombosis of small vessels was noted in many studies [17,24,25,26]. 

Cytotoxic endothelial dysfunction was considered a contributing mechanism. 5-FU reduces the antioxidant capacity, enhancing the generation of free radicals and resulting in lipid peroxidation and early atherosclerosis [26,27]. Moreover, 5-FU induces changes in the morphology and metabolism of red blood cells and decreases their ability to transfer oxygen. All these changes impair the myocardial oxygenation and lead to cardiomyocyte ischemia [28]. 

Although initially considered less cardiotoxic than 5-FU, capecitabine was related to acute coronary events as well. The spasm of the coronary artery was considered the underlining substrate [29,30]. The incidence of cardiotoxicity of capecitabine varies between 3% and 9%, which is similar to that of continuous 5-FU infusion therapy [31]. Gemcitabine rarely induces ACSs, but when they do occur, they are the most acute MI. Patients receiving gemcitabine in combination with another anticancer drug are at the highest risk [20]. 

The timing from drug administration to acute coronary event onset is highly variable. Coronary artery spasm may manifest during infusion or hours or days after drug administration [20,32,33,34]. The explanation for this phenomenon is still elusive. 

Coronary spasm is the main expression of the vascular damage caused by FP. Therefore, FP must be discontinued immediately, and the treatment with low-dose aspirin, calcium channel blockers (CCBs), and long-acting nitrates should be initiated. The efficacy of non-dihydropyridine CCB, such as diltiazem and verapamil, and of nitrates was reported in different clinical scenarios. One patient with transient ST-segment elevation ACS and ventricular tachycardia was temporarily treated with diltiazem 180 mg/day, while capecitabine was definitively withdrawn. Low-dose aspirin and an inhibitor of the renin-angiotensin system, telmisartan, were also recommended [35]. Another therapeutic option is to administer a bolus of diltiazem and an intravenous infusion of nitroglycerin, followed by isosorbide mononitrate 40 mg/day and diltiazem 90 mg/day [36]. In both cases the troponin and coronary angiography were normal, reinforcing the idea that coronary spasm was the underlining substrate. Antianginal therapy with nitrates and non-dihydropyridine CCB allowed symptoms control in up to 70% of patients [31]. 

However, the prevention of coronary spasm using CCB and/or nitrates showed mixed results. While diltiazem and nifedipine have accumulated positive evidence, verapamil failed to prevent 5-FU-induced vasospasm [37,38,39].

Cardiac ischemia related to the use of FP is associated with an 82 to 100% risk of symptom recurrence at the reintroduction of the drug and up to 13% risk of death; therefore, further treatment with these compounds should be discouraged [40]. If mandatory, i.e., the best chance for survival or lack of other therapeutic alternatives, CT may be restarted under protection from CCB and nitrates [35]. Drug re-challenge include short-acting diltiazem and sublingual nitroglycerin as needed during FP infusion and pre- and post-treatment with extended-release nifedipine and isosorbide mononitrate [41].

Endothelial damage leads to microthrombosis, and experimental studies suggested that the use of anticoagulants may partially mitigate this toxicity. The protective role of a low-molecular-weight heparin against the prothrombotic effect of 5-FU on the vascular endothelium was assessed in an animal model [25]. Dalteparin reduced the burden of thrombosis, but the reversibility of the endothelial damage was impaired. It was concluded that dalteparin offers protection from the thrombogenic effect of 5-FU, but this anticoagulant per se had a toxic effect on the endothelium, which was different from that of 5-FU. 

The efficacy of probucol—a lipid-lowering drug with strong antioxidant properties—was assessed in the prevention of 5-FU toxicity on vascular endothelium, and it showed promising results. The damage to the endothelium was minimal and comparable to that of the control group [26]. 

## 3. Platinum Compounds (Cisplatin)

Cisplatin-based CT is widely used for the treatment of many solid-organ cancers. Still, its high efficiency is accompanied by multiple toxicities: ototoxicity, renal, digestive, cardiovascular, pulmonary toxicities, myelosuppression, and allergic reactions [42]. One of the manifestations of cardiovascular toxicity is arterial and venous thrombotic events. Although rare, any of the following have been reported: angina pectoris, acute MI, transient ischemic attack, stroke, and deep vein thrombosis. The overall incidence of thrombotic complications is 10% and less in the arterial system (1.6%) than in the venous system (8.4%) [43]. Acute cardiovascular complications usually occur during treatment, but cisplatin-based CT increases the risk of acute vascular events in the long term as well. 

Cisplatin has direct and acute vascular toxicity. It inhibits the proliferation of endothelial cells, induces their apoptosis, and thereby reduces their survival [44]. Cisplatin increases the generation of reactive oxygen species and triggers an inflammatory response with cytokine release. The oxidative stress and inflammation intensify each other and lead to endothelial dysfunction. As a consequence, the nitric oxide (NO) levels decrease leading to a prolonged vasoconstrictor response. Moreover, a prothrombotic environment is created, characterized by increased platelet activation and high levels of von Willebrand factor, fibrinogen, and tissue-type plasminogen activator. The activity of natural anticoagulants is impaired, as evidenced by decreased functional protein C levels. 

Cisplatin also indirectly contributes to increased cardiovascular risk. Its ability to generate reactive oxygen species leads to increased lipid peroxidation. It disrupts the lipid metabolism leading to proatherogenic dyslipidemia [45,46]. Total cholesterol, LDL-cholesterol, total cholesterol/HDL cholesterol ratio, and triglycerides were significantly higher in patients receiving CT compared with those treated only with surgery. In one study, the prevalence of hypercholesterolemia was as high as 80% [47]. Long-term observational studies showed that cisplatin can induce hypertension and insulin resistance. The overall prevalence of diabetes was 7.3% and was the highest in patients who received both RT and CT. It was four times higher than in patients treated with surgery alone. Moreover, metabolic syndrome was more common in patients who received CT than in those who did not [48]. 

The patients treated with cisplatin-based CT have a significantly increased risk of major coronary events, especially during and shortly after drug administration. Intravascular thrombosis is considered the main underlining substrate. Cisplatin may induce arterial thrombosis with subsequent myocardial and cerebrovascular ischemia in 2% of patients [49]. While in the general population, a complicated atherosclerotic plaque is responsible for the acute MI, in patients treated with cisplatin, arterial thrombosis occurs even in the absence of underlying atherosclerosis or classical cardiovascular risk factors. In a 71-year-old woman presenting with ACS, the coronary angiography showed a subtotal thrombotic occlusion of the proximal segment of anterior descending artery (ADA) and embolic occlusion of the distal segment of ADA but no significant coronary stenosis [50]. Similarly, in a 31-year-old man without cardiovascular risk factors presenting with ST-elevation myocardial infarction (STEMI) 24 h after CT, the coronary angiography performed the day after intravenous thrombolysis showed moderate mid-ADA disease with residual thrombosis [51]. In another young male presenting with STEMI, extensive left coronary thrombosis was identified, with the thrombotic load of the circumflex being more important than that of the ADA [52].

Vasoconstriction is considered an important contributor to the ACS, especially when the patients are very young, there are no cardiovascular risk factors, or the coronary angiography is normal [45,53]. In a 34-year-old man with no cardiovascular risk factors presenting with STEMI 5 days after CT, the coronary angiography revealed mild stenosis at the level of the right coronary artery with overlying thrombus and vasospasm and no other significant coronary lesions [54]. Two cases of vasospastic angina and one case of acute MI secondary to vasospasm have also been reported in connection with ongoing cisplatin CT [55,56]. Because 70–80% of patients treated with cisplatin develop hypomagnesaemia, and this can induce vasospasm, in the latter case, a possible role for this electrolyte imbalance has been considered as a stimulator of vasospasm.

Spontaneous coronary artery dissection during cisplatin therapy is extremely rare [57]. In a 33-year-old man who presented STEMI at the end of the first cycle of CT, coronary angiography revealed a circumscribed stenosis of the circumflex artery with intima dissection and associated thrombosis and no atherosclerotic plaque [58]. In another young patient, a chronic dissection of the right coronary artery was identified at the angiography. 

In 5.7–6.7% of cisplatin-treated cancer survivors, CAD manifests more than a decade after CT [59]. The absolute risk of CAD is up to 8% over 20 years [9]. At 30-year follow-up, the same population had more CAD and was more frequently treated for hypertension and dyslipidemia than controls [60]. Data from a Norwegian study showed that the risk of CAD is 2.6 times higher in patients receiving cisplatin-based CT and 4.8 times higher in those receiving CT and RT than in patients treated only by surgery [9]. In an older study, the risk of CAD, reported as non-fatal MI and angina pectoris with proven myocardial ischemia, was 7 times higher in patients receiving cisplatin than in the general population [47]. Because these data come from studies that have enrolled patients with germ cell tumors, which most commonly affect men between the ages of 15 and 35, it can be concluded that cisplatin treatment induces accelerated atherosclerosis and premature CAD [61]. It was observed that intima-media thickness is higher than the estimated value for age, confirming that cisplatin triggers a degenerative process of the vessel walls, leading to occlusive vascular disease in the long term [62]. Moreover, cisplatin has the potential risk of delayed onset of vasospastic angina [63].

Cisplatin therapy is associated with oxidative stress and increased reactive oxygen species production. Moreover, an increase in oxygen free radicals also exists during the reperfusion of ischemic cardiac tissue. Therefore, inhibiting this pathway can be beneficial. In an animal model, the administration of *N*-Acetyl-l-cysteine—a free radical scavenger—ameliorated the coronary flow and alleviated the effects of cisplatin [64]. In preclinical studies, acetyl-carnitine, α-lipoic acid, and silymarin have also been shown to have antioxidant potential, limiting the cardiac toxic effects of cisplatin [65].

Coronary vasospasm during treatment may lead to decreased dose or discontinuation of cisplatin and a more unfavorable oncological prognosis for the patient. The decision to withdraw cisplatin after acute MI due to coronary spasm was reported [56]. The CCB and long-acting nitrates effectively prevent coronary spasm and currently constitute the treatment of choice of vasospastic angina in the general population [66]. A CCB was initiated in a patient with ACS and normal angiography, with cisplatin-induced coronary spasm as a substrate. Under this protection, the course of CT was without further events [53]. This strategy is in line with current guideline recommendations [66]. However, routine administration of low-dose aspirin or lipid-lowering treatment in patients with normal angiographic coronaries is not recommended [67]. 

Cisplatin-based CT is often followed by the onset of metabolic syndrome [48,68,69], which will further increase the risk of major coronary events. Therefore, aggressive therapeutic interventions aimed at correcting the components of the metabolic syndrome should be a priority. In the absence of specific recommendations, the therapeutic intervention consisting of lifestyle interventions and drug therapy mimics that in the general population [70]. One study explored the utility of tailored exercise interventions to ameliorate cardiovascular dysfunction in patients with a history of cisplatin CT, highlighting the favorable metabolic impact [71]. 

## 4. VEGF/VEGFR Inhibitors (Bevacizumab, Sorafenib, Sunitinib)

Vascular endothelial growth factor (VEGF) pathway inhibitors are drugs used to treat a wide variety of cancers due to their inhibitory effect on angiogenesis. The most extensive experience comes from advanced renal cell carcinoma patients in whom the treatment increased the overall survival rate [72]. VEGF pathway inhibitors have also been used successfully in treating non-small cell lung cancers, hepatocellular carcinomas, pancreatic neuroendocrine tumors, gastrointestinal stromal tumors, colorectal, breast, ovarian and thyroid cancers, and glioblastoma [73,74,75,76,77,78,79,80]. 

Bevacizumab is a recombinant humanized monoclonal antibody that binds to VEGF and blocks the interaction between VEGF and its receptor on the surface of endothelial cells. As a result, endothelial cell proliferation and the formation of new blood vessels are inhibited [81]. Sunitinib and sorafenib are tyrosine kinase inhibitors for VEGF receptor (VEGFR) and act by inhibiting the VEGF pathway. 

While inhibiting angiogenesis is a therapeutic advantage, the decreased endothelial cell regeneration capacity leads to the formation of areas of endothelial discontinuity, where exposed subendothelial collagen interacts with tissue factor and initiates coagulation. VEGF inhibition diminished NO production as well, thus impairing vasodilation. Since NO prevents leukocyte and platelet adhesion to endothelial cells and platelet aggregation and stimulates disaggregation of preformed platelet aggregates, its decreased production leads to a prothrombotic state [82]. Moreover, bevacizumab increases the expression of proinflammatory cytokines, enhancing thrombosis. 

Up to 4–5% of patients receiving bevacizumab will develop arterial thrombotic events during treatment, with the highest risk being recorded in patients with metastatic cancer and in the first 3 months of therapy [40,83]. Adding bevacizumab to CT will double the risk of stroke, MI, coronary heart disease, and cardiac death. When only coronary events were assessed, bevacizumab increased the incidence of MI/angina pectoris from 1% to 1.5% [84]. In clinical studies, the incidence of CAD ranged from 0.52% to 1.7%. A meta-analysis of seven studies evaluating the effect of bevacizumab on the occurrence of CAD in cancer patients showed that the risk was 2.5 higher compared to the general population. The risk was not evenly distributed between tumor types, with patients with colorectal cancer having the highest risk [81]. A larger meta-analysis showed that adding bevacizumab to standard CT increases the overall risk of cardiac ischemia by 2.47 times. In patients receiving a high dose of bevacizumab regimen, the risk was 4.4 times higher [85]. Age over 65 years and a positive history of CAD/atherosclerosis were identified as risk factors for arterial thrombosis [84]. 

The incidence of arterial thrombotic events is 1.7% for sorafenib and 1.4% for sunitinib [86]. In addition, the treatment with sorafenib or sunitinib increases three times the risk of arterial thrombotic events regardless of the drug used or the cancer type. In patients with advanced clear-cell renal-cell carcinoma, the treatment with sorafenib increased the incidence of cardiac ischemia or MI from less than 1% to 3% (*p* = 0.01) [87]. In one case acute MI was recurrent at four and five years after initiation of sorafenib [88]. 

A large meta-analysis of 23 trials showed that anti-VEGF agents significantly increased the risk of severe arterial thrombotic events [89]. Major arterial thrombotic events—mostly involving the coronary arteries—affected one in 27 patients receiving bevacizumab [90]. In general, 1–15% of patients treated with an inhibitor of the VEGF pathway will report chest pain episodes, ranging from stable angina to ACS [91]. Cardiac events are still underestimated in patients receiving either sorafenib or sunitinib. One study reported that 33.8% of patients experienced a cardiac event. Still, after adequate cardiovascular management, all patients continued the treatment with tyrosine kinase inhibitors [92].

Hypertension is the most frequent complication during bevacizumab treatment, occurring in one of three patients. The risk of hypertension is increased three times with a low dose and 7.5 times with a high dose of bevacizumab [93]. Sorafenib and sunitinib are associated with an increased risk of hypertension as well. One in four patients receiving sorafenib and one in five patients receiving sunitinib will develop abnormal blood pressure values [94,95]. Hypertension leads to endothelial dysfunction and may enhance the vascular toxicity of VEGF pathway inhibition. 

Bevacizumab treatment is associated with an increased risk for bleeding, which is usually minor and not requiring intervention. Severe bleedings occur in less than 5% of cases [96]. When added to CT, sorafenib or sunitinib double the risk of bleeding, but the incidence of severe bleeding was raised only slightly, probably due to the limited sample size and events [97]. Therefore, in patients with cancer under CT that have an increased risk of both thrombosis and bleeding, thromboprophylaxis is a challenge. 

Baseline cardiovascular risk assessment and optimal control of arterial hypertension are general measures aiming to reduce the risk for acute coronary events, especially when evidence-based oncology treatments have potential cardiovascular toxicity. Risk assessment provides the opportunity for early intervention on modifiable cardiovascular risk factors to properly diagnose and treat cardiovascular disease in order to reduce the risk of cardiovascular complications during and after cancer treatment. The risk scores for the general population do not fully reflect the cardiovascular risk of neoplastic, so baseline risk stratification proformas have been specifically developed for patients scheduled to receive medication known to be cardiotoxic, including VEGF pathway inhibitors [98].

Although hypertension has a multifactorial substrate, the role of NO depletion should be emphasized. NO regulates the renal blood flow and tubular sodium excretion. Low NO levels lead to impaired sodium excretion and consequently fluid retention and salt-dependent hypertension [99]. Therefore, diuretics and NO donors such as nitrates should be considered [100]. CCB are potent vasodilators. Only nifedipine and amlodipine are allowed because they do not have the adverse effect of increasing VEGF levels as do verapamil and diltiazem, which are therefore to be avoided [79]. Of beta-blockers, carvedilol is the best choice, as it has both vasodilatory and antiangiogenic effects. Although nebivolol has an appropriate mechanism of action, i.e., it increases endothelial NO production, it is not preferred, as it may counteract the inhibitory effect of anticancer drugs in tumor vessels [101]. The renin-angiotensin system is less important in the mechanism of hypertension induced by the treatment with VEGF pathway inhibitors [102]. Still, angiotensin system inhibitors added benefits to cancer treatment, leading to superior survival outcomes for patients with metastatic renal cell carcinoma. In light of this evidence, angiotensin system inhibitors—with or without amlodipine—are the first choice in patients without comorbidities that require the use of other pharmaceutical classes. Carvedilol is the third step, followed by diuretics. Long-acting nitrates are reserved for resistant hypertension [79]. Optimal blood pressure goals are not defined at present; therefore, those valid for the general population apply. Still, due to their high cardiovascular risk, some authors have proposed lower targets in cancer patients [79,103]. 

There is evidence for and against the use of statins. Statins have pleiotropic effects and can ameliorate endothelial dysfunction. However, by improving NO bioavailability and eNOS activity, they could reduce the efficacy of VEGF pathway inhibitors. Statins reduce VEGF expression in microvascular endothelial cells and enhance it in macrovascular endothelial cells. Moreover, statins are proangiogenic at low doses and antiangiogenic at high doses [104]. Due to this duality, when given to cancer patients, high doses of statins are recommended.

Because a large number of patients, in some studies up to 20–40%, experience bleeding during cancer treatment with VEGF pathway inhibitors, the use of antiplatelet and anticoagulant therapy is of major concern, especially when intended for primary prevention [96]. Low-dose aspirin use slightly reduced the rate of arterial thrombotic events in patients treated with bevacizumab. The effect was more important in the subgroup of patients over the age of 65 and with a history of arterial thromboembolism [84]. Still, no conclusion could be drawn due to the small number of events per risk factor subgroup. No significant differences in the risk of major bleeding were found between low-dose aspirin users and non-users among control and bevacizumab-treated patients. In a non-randomized study, the rate of arterial thrombotic events in patients treated with bevacizumab was 2.7% in non-low-dose aspirin users and 8.9% in low-dose aspirin users [90]. Once again, no definitive conclusions could be reached regarding any preventive effects associated with low-dose aspirin use. The interpretation of the data was hampered by a higher proportion of patients with cardiovascular risk factors or a previous arterial thrombotic event among the low-dose aspirin users. 

The benefits of low-dose aspirin in CAD patients in the general population are undisputed [105]. Therefore, antiplatelet therapy should be continued during the treatment with VEGF pathway inhibitors in patients with a diagnosis of CAD because the potential benefits outweigh the risk of bleeding complications [106,107]. However, patients without CAD should not receive antiplatelet drugs as part of primary prevention. Moreover, it is recommended to discontinue bevacizumab for any arterial thrombotic event. 

## 5. Radiotherapy

Chest RT is an essential part of the treatment of hematological malignancies and thoracic solid tumors, such as lung, esophageal, and breast cancers. Due to close proximity to the irradiated area, the heart and coronary arteries receive variable amount of radiation. Any of the heart structures can be affected, leading to constrictive pericarditis, CAD, myocardial disease, valvular heart disease, and conduction system dysfunction. Radiation-induced CAD (RICAD) is a late complication of RT. The most vulnerable patients are those with Hodgkin’s lymphoma and women undergoing treatment for left-sided breast cancer [108,109,110]. RICAD is the second most common cause of morbidity and mortality in patients with RT for thoracic malignancies, especially breast cancer and Hodgkin’s lymphoma, due to the favorable long-term prognosis and/or relatively young age of patients. The time between RT and RICAD onset may be short—a year or two—if the radiation dose was high, i.e., above 30–35 Gy, or traditional atherosclerotic risk factors were present, but more than a decade could elapse if lower doses were used [111,112,113,114]. RICAD occurs more than 10 years from the completion of treatment in patients with breast cancer [115] and after two or three decades post-therapy in patients with Hodgkin’s lymphoma [116].

During the irradiation of the left breast and internal mammary chain, the heart receives a radiation dose of 0.9–14 Gy and 3–17 Gy, respectively. Lower doses of 0.4–6 Gy and 2–10 Gy, respectively, are received by the heart during the irradiation of the right breast and internal mammary chain [117]. This difference is important because it results in a higher prevalence of RICAD and MI in women receiving RT for left compared to the right breast cancer [118]. Moreover, RT increases the cardiac mortality in women with breast cancer [119]. Although cardiac irradiation is less intense with current modern techniques, the mean dose to which the heart is exposed varies between 1 and 5 Gy [113,120,121]. It must be emphasized that the radiation dose received by the ADA is greater than the whole heart dose; therefore, the risk of developing ischemic heart disease remains. The risk of a major coronary event, e.g., MI, coronary revascularization, or death from ischemic heart disease, increases linearly with the mean dose of radiation to the heart over several decades, with 7.4% per Gy, whether or not there are pre-existing cardiovascular risk factors [113]. There was no threshold below which there was no risk. The risk of major cardiovascular events doubles in the presence of cardiovascular risk factors and increases more than 6 times in the case of an ischemic heart disease history [113,122,123]. Up to 3- to 4-fold increase in the risk of MI due to CAD has been observed if mediastinal irradiation was combined with CT [124].

RICAD is highly prevalent in Hodgkin’s disease survivors and brings significant morbidity and mortality. The most robust evidence of coronary damage due to RT comes from studies on pediatric population [125,126]. Mediastinal RT during childhood is significantly correlated with the presence of coronary artery abnormalities on computed tomography angiography (CTA) and is associated with a 4 to 6 times increase in the risk of CAD compared to the general population [108]. In asymptomatic patients, the risk of RICAD is 16% in the first 10 years, and it is dependent on the radiation dose, being 6.8 times higher in those who received more than 20 Gy compared to those who received lower doses [127]. The need for coronary artery bypass grafting (CABG) and percutaneous coronary intervention (PCI) is 3.2 times and 1.6 times higher, respectively, than in the general population [128], and MI is the most common cause of cardiac death in these patients. Severe fibrotic and calcified stenosis of the proximal part of the left main (LM) was identified during autopsy in a 12-year-old boy with MI six years after chest RT [129]. 

RT leads to both macrovascular and microvascular disease. The evolution of atheroma plaques in these patients mimics that of the general population, but the process is accelerated, and the amount of fibrosis is higher [115,130]. Radiation injures the endothelial cells and disrupts their membranes. It also triggers the activation of inflammatory pathways, platelets, and coagulation cascade, leading to thrombosis. The endothelium becomes porous, releases inflammatory mediators, and expresses adhesion molecules that facilitate the passage of low-density lipoproteins and monocytes in the subintimal space [131]. The formation of foam cells sustains chronic inflammation that ultimately leads to atherosclerotic plaque instability and rupture, diffuse fibrosis of all layers of the coronary artery wall, and intimal hyperplasia [116,132,133,134]. Endovascular thrombosis and capillary luminal stenosis determine a reduction in capillary density [135]. The endothelial dysfunction is responsible not only for impaired vascular reactivity, e.g., coronary spasm or persistent vasoconstriction, but also acts synergistically with sustained inflammation to maintain the prothrombotic environment [136]. 

Typical for RICAD are ostial stenoses and severe atherosclerotic lesions on the proximal segments of the epicardial coronary arteries. The LM trunk and the proximal segments of the ADA and right coronary artery are mainly affected. Due to their anterior and central position in the mediastinum, they receive higher doses of radiation compared to the lateral, posterior, and peripheral coronary vessels. The atherosclerotic plaques are long, smooth, fibrotic, and cause concentric and tubular narrowing of the vessel lumen. Negative remodeling is frequently encountered [116]. Long segments of diffuse disease and areas of stenosis from soft plaque were also found [126]. Microvascular fibrosis and reduction of capillary density lead to dysfunction in microcirculation, reduction of the coronary flow reserve, and, finally, to myocardial ischemia [3]. Acute coronary events may occur, as RT can trigger coronary spasm and atherosclerotic plaque rupture, followed by partially or totally obstructive thrombosis [137].

The deleterious effects of RT on heart structures are well-known, and over the last decades, continuous efforts have been made to limit them. Many improvements to RT protocols have been added, such as enhanced localization and gated techniques. Three- and four-dimensional planning models and those based on positron emission tomography/X-ray computed tomography are widely implemented in practice [135,138]. For patients with left-sided breast cancer, intensity-modulated RT and deep inspiration breath-hold (DIBH) technique are also used to reduce the harmful effect of radiation [139,140,141,142]. A reduction in the maximum and mean dose received by the ADA or by the whole heart was obtained in several studies evaluating the DIBH technique, but this did not apply to all patients, emphasizing the importance of individual anatomical chest features [140,143,144,145,146]. Nowadays, proton therapy—the most precise form of radiation treatment available—is gaining more and more ground in front of photon therapy [147].

Although the most important prophylaxis of IRCAD is to reduce the dose of radiation to the heart and coronary arteries—current RT protocols strive to achieve this—some exposure remains inevitable. Therefore, pharmacologic cardio-protective interventions have been considered as well, mainly focusing on limiting oxidative stress, inflammation, fibrosis, and thrombosis [147].

Statins are drugs with multiple effects. They have lipid-lowering properties, reducing cholesterol and lipoprotein density by inhibiting 3-hydroxy-3-methylglutaryl coenzyme A reductase. However, the mechanism of action of statins exceeds the metabolism of lipids. Their pleiotropic effects are numerous and encompass vascular tone improvement and anti-inflammatory, antithrombotic, antifibrotic, and oxidative stress-reducing properties [148]. The benefit of statin treatment on the atherosclerotic plaque is unquestionable. The inflammation within the atherosclerotic plaque is reduced [149], and the composition and volume are altered, leading to plaque stabilization [150] and even regression [151]. One of the strengths of statins is that they can prevent endothelial damage. Since this is the main trigger of the complex pathophysiological processes leading to both atherosclerosis and fibrosis, statin treatment can hinder the development and progression of atherosclerotic plaques, acting from the early phases of this process [152]. 

In animal and cell culture models, statins ameliorated radiation-induced inflammation and fibrosis in different tissues [153,154,155]. When their effect on the heart and blood vessels was specifically assessed, atorvastatin ameliorated radiation-induced cardiac fibrosis, prevented vascular damage, reduced endothelial cell apoptosis, and promoted the healing of the radioactive injuries [124,156,157]. Nevertheless, the association between atorvastatin and an anti-platelet drug, i.e., clopidogrel, failed to inhibit either age-related or radiation-induced atherosclerosis [158]. Lovastatin prevented endothelial cells from radiation-induced cell death [159]. Simvastatin partially mitigated the radiation-induced fibrosis of the penetrating coronary vessels, the severity of MI, and the increase in low-density lipoprotein levels [160]. Pravastatin had anti-inflammatory and antithrombotic effects on endothelial cells [161]. It has the potential to reduce radiation-induced endothelial dysfunction and limit the leukocyte and platelet’s adhesion to endothelial cells [162].

Although there is abundant evidence of the beneficial effect of statins in reducing cancer-related and overall morbidity and mortality after RT for thorax, head, and neck cancers [135], the direct assessment of the radio-protective role of statins on the cardiovascular system is still very limited. The main data come from Boulet et al.’s study, which proved that statin treatment provided protection against vascular events by reducing the composite endpoint of stroke, MI, or death caused by stroke or MI [4]. It should be noted that the patients enrolled were over the age of 65, with a history of coronary angiography, ACS, or coronary revascularization, and therefore, the results may be blunted by the high-risk population included. Complementary data are provided by the study of Addison et al. that enrolled younger patients with fewer comorbidities undergoing RT for head and neck cancers [163]. Patients treated with statins during RT had a 60% reduction in the incidence of ischemic stroke or TIA, which confirmed the benefit of statin therapy in reducing the risk of cerebrovascular events. Since no randomized clinical trials offer to date an assessment of the clinical impact of statins on outcomes in radiation-induced vascular disease, this result allows us to only assume that statins could have a similar radio-protective effect on the coronary arteries. 

Low-dose aspirin is a widely prescribed drug that can block platelet aggregation, reduce inflammation, and prevent thromboxane A2-induced vasoconstriction. In the general population, all the evidence supports the usefulness of low-dose aspirin in the secondary prevention of cardiovascular disease [105]. In individuals without atherosclerotic disease, more evidence is needed. Still, there is consensus that low-dose aspirin should not be given routinely but only in patients with high or very high cardiovascular disease risk, taking into consideration both ischemic and bleeding risks. In preclinical studies, aspirin showed a radioprotective effect in different tissues [135] but not on the vascular bed [164]. Both nitric oxide-releasing aspirin and aspirin attenuated age-related atherosclerosis but failed to reduce radiation-induced atherosclerosis. In clinical studies, low-dose aspirin registers the overwhelming evidence to reduce cancer mortality [165]. Still, there are no clinical trials to specifically evaluate the role of low-dose aspirin in the modulation and prevention of RICAD. 

Angiotensin-converting enzyme inhibitors have multiple effects on endothelial cells. They reduce the endothelial production of angiotensin II and therefore limit vasoconstriction, reduce levels of adhesion molecules and growth factors, decrease oxidative stress, and prevent apoptosis. They also decrease the degradation of endothelial bradykinin, thus leading to vasodilation by stimulating the production of nitric oxide and other relaxing factors [166]. Angiotensin II receptor blockers limit vasoconstriction by selectively inhibiting the AT1 receptor and exert antiplatelet, anti-inflammatory, and antimitogenic effects independently of action on the AT1 receptor [167]. Angiotensin II levels increase locally after irradiation and contribute to inflammatory responses, vascular damage, and fibrosis [168], and as such, limiting these effects could be beneficial. Captopril is the angiotensin-converting enzyme inhibitor with the most evidence of reducing radiation damage in various tissues, most likely due to the sulfhydryl group with the activity of free radical scavenger. Angiotensin II may initiate cardiac perivascular fibrosis, but captopril administration was associated with reduced early radiation-induced cardiac fibrosis [169]. Moreover, captopril exerted inhibitory effects on the TGF-β1-mediated pathway leading from endothelial dysfunction to fibrosis [170]. Preclinical studies showed that AT1-receptor blockade mediated similar radioprotection as the ACE inhibitor [171,172,173], but there are no data from clinical trials. 

Colchicine has anti-inflammatory properties with a mechanism of action independent of the arachidonic acid pathway and reduces thrombin-induced platelet aggregation [174]. It impairs leukocyte mobility and inhibits neutrophil infiltration into the intima. Hence, there are two major advantages: it decreases the risk of atherosclerotic plaque destabilization and mitigates the fibrotic process, as neutrophils are their major contributors [175]. In clinical trials, colchicine reduced the risk of MI in patients with CAD [176], and based on these encouraging results, low-dose colchicine may be considered in selected high-risk patients with the established atherosclerotic disease [105]. The possibility of using colchicine in the prevention of RICAD has been highlighted based on preclinical data and results from trials in patients with CAD in the general population [135,177,178]. However, there are no clinical trials specifically evaluating colchicine in the prevention of RICAD.

Despite the presence of data showing the association between RT and vascular disease, be it additive or multiplicative to traditional cardiovascular risk factors, no guidelines currently exist for the treatment or prevention of radiation-induced atherosclerosis. Risk factor modification appears to have the greatest potential for reducing RICHD risk though it has not been prospectively studied and should begin prior to RT.

## 6. Discussion

The burden of cancer incidence and cancer-related deaths is growing rapidly worldwide. The International Agency for Research on Cancer has announced that 19.3 million new cancer cases and 10.0 million cancer-related deaths occurred in 2020 [179]. Breast, lung, colorectal, prostate, and gastric cancers are the malignancies with the highest global incidence, accounting for 11.7, 11.4, 10, 7.3, and 5.6% of all newly diagnosed cancers, respectively. In men, lung cancer ranks first (14.3%), followed by prostate (14.1%), colorectal (10.6%), and gastric cancers (7.1%). Breast (24.5%), colorectal (9.4%), and lung cancers (8.4%) are the most common newly diagnosed malignancies in women. In the last decade, in many countries, the prevention and treatment of cardiovascular disease have intensified, leading to a marked decrease in the mortality rate caused by stroke and CAD relative to cancer, which has now positioned cancer as the leading cause of death in the world. Lung cancer is responsible for 18% of all cancer-related deaths. In terms of mortality, it ranks first in men and second in women after breast cancer [179]. 

In the last 30 years, more than 150 new molecules have been introduced into the treatment of cancer. Platinum-based anticancer drugs, anti-angiogenesis agents, and anthracyclines are among the most prescribed anticancer drugs today [180,181,182]. Moreover, platinum-based anticancer drugs are used as components of almost half of all cancer treatments. Among anti-angiogenesis agents, those targeting the VEGF pathway are by far the most commonly used. FPs are currently the third most commonly used anticancer drug in the treatment of solid cancers, such as colorectal and breast cancer [183]. RT is frequently used in combination with surgery or systemic CT, and more than half of patients receive RT as part of their cancer treatment. 

Due to the high frequency of cancers globally and the widespread use of potentially cardiotoxic antineoplastic drugs, the possibility of cardiovascular adverse events during evidence-based oncological treatments should be considered. Many cancers are diagnosed after the age of 50, when cardiovascular risk factors such as high blood pressure, diabetes mellitus, dyslipidemia, obesity, and smoking may be present, which can exacerbate the cardiovascular toxicity of some anticancer drugs [184]. 

Cancer treatments induce endothelial dysfunction, spasm of the coronary arteries, thrombosis, and fibrosis. The effort to mitigate these harmful pathophysiological processes is hampered by the lack of randomized studies to provide robust and reliable data. Although unitary as a concept, all starting from the pathophysiological triad aforementioned, the therapeutic lines of prevention that have been outlined so far have a number of individual features determined by the mechanism of action of the anticancer drug (Table 1).

Vascular injury may have clinical expression during or shortly after treatment, as in the case of oncological therapy that induces vasospasm or thrombosis, or it may develop asymptomatically for a long time, as in the case of atheroma plaque formation and growth. The symptoms may occur months and sometimes years following the completion of CT. Factors attributable to the drug, such as type of drug, cumulative or total dose, and schedule, along with individual factors influence the incidence and severity of CT-induced adverse cardiac effects [20]. 

For other anticancer drugs, namely anthracyclines, the hypothesis of impaired coronary circulation as a contributor to their cardiac toxicity is gaining increasing interest. Although they have been in use for more than half a century and are the anticancer drugs the most associated with CT-induced cardiac toxicity, the exact mechanism by which heart damage occurs is not yet fully elucidated [182,185]. Anthracyclines’ main adverse cardiac effect is overt heart failure or a drop in the left ventricular ejection fraction [186]. There is indirect evidence of the toxic effect of anthracyclines on coronary arteries. In cultured cells, doxorubicin and daunorubicin caused endothelial cell dysfunction by increasing oxidative stress in the vessel wall [187,188]. Moreover, anthracyclines induced apoptosis in smooth muscle cells [189] and increased the thickness of media, adventitia, and total coronary arteriolar wall [190]. Recently, in an animal model, it was shown that anthracyclines produced irreversibly damage to the coronary microcirculation [191], and in patients with lymphoma, defects were identified in myocardial perfusion by positron emission tomography after a single doxorubicin dose [192]. Therefore, in-depth research on the effect of oncological therapy on coronary arteries is expected to provide clear answers in the future for many of the current unknowns related to the cardiac toxic effects of anticancer drugs. 

Several practical aspects must be retained. Firstly, the cardiovascular risk factors must be identified since they are important contributors to the progression of atherosclerosis and the onset of acute coronary events. This is of major concern since studies of cancer survivors beyond 5 years post-diagnosis have demonstrated a 1.7- to 18.5-times increased incidence of cardiovascular risk factors including hypertension, diabetes mellitus, and dyslipidemia when compared with age-matched counterparts without a history of cancer [193]. Lifestyle changes such as smoking cessation and exercise and pharmacological therapies are similar to those in the general population although some authors have proposed stricter targets [79,103]. Baseline risk stratification proformas are currently available and allow clinicians to stratify cancer patients into low-, medium-, high-, and very-high-risk categories of cardiovascular complications prior to starting treatment and whenever needed during treatment. The ultimate goal is to provide personalized approaches to minimize the risk of cardiovascular toxicity from cancer therapies [98].

Secondly, there are no randomized clinical trials specifically dedicated to the study of prevention measures to support any specific strategy. The available data come from studies evaluating specific oncological therapies in specific types of cancers, so a general conclusion is far from drawn. However, there is a benefit the existence of a sufficient amount of evidence to allow some modulation and customization in cancer patients of the treatments indicated in the general population, including medical therapy, PCI, and CABG.

Although based on an extensive search, our study has several limitations. Firstly, there was no universal definition of the cardiovascular toxicities of anticancer drugs until earlier this year, when an international consensus statement defined the cardiovascular toxicities of cancer therapies [194]. In the already published literature, the term includes the myocardial, pericardial, endocardial, and conduction systems and CAD due to cancer treatments, which made it difficult to select only the information related to coronary arteries. Moreover, many studies reported acute thromboembolic events as a sum of coronary and cerebral arterial events. Secondly, there are many therapeutic agents used in the treatment of cancers and a huge amount of literature published so far, sometimes with conflicting data, which made it difficult to interpret them. Moreover, some drugs have recently been introduced in practice and therefore offer little or no data on the effects on the coronary arteries.

## 7. Conclusions

Antineoplastic therapy saves or at least prolongs life, but this is achieved at the cost of major side effects, such as cardiovascular ones. CAD and especially acute MI have the potential to limit the benefits of cancer treatment. Our work provides the most comprehensive, systematized, structured, and up-to-date analysis of the available literature, focusing on measures aiming to protect the coronary arteries from the toxicity of cancer therapy. This approach facilitates their implementation in daily practice.

## Figures and Tables

**Table 1:** Mechanisms of CAD induced by cancer treatment and proposed interventions.

Drug	Mechanism	Intervention	References
Antimetabolites (5-FU, Capecitabine, Gemcitabine)	Coronary artery spasm Intravascular thrombosis Endothelial injury	Immediate discontinuation of the drug Calcium channel blockers Long-acting nitrates Short-acting nitrates (on demand) Low-dose aspirin Dalteparin ^b^ Probucol ^b^	[25,26,31,35,36,37,38,39,41]
Platinum Compounds (Cisplatin)	Intravascular thrombosis Coronary artery spasm Coronary artery dissection Endothelial injury Accelerated atherosclerosis Proatherogenic dyslipidemia Hypertension Insulin resistance	Decreased dose or discontinuation of the drug Calcium channel blockers Long-acting nitrates Low-dose aspirin ^a^ Statins ^a^ *N*-Acetyl-l-cysteine ^b^ Acetyl-carnitine ^b^ α-lipoic acid ^b^ Silymarin ^b^	[53,56,64,65,67]
VEGF/VEGFR inhibitors (Bevacizumab, Sorafenib, Sunitinib)	Intravascular thrombosis Endothelial injury Hypertension	RAS inhibitors Calcium channel blockers (only DHP) Beta-blockers (only carvedilol) Diuretics Long-acting nitrates Statins Low-dose aspirin ^a^	[79,84,90,96,100,101,102,104,106,107]
Radiotherapy	Accelerated atherosclerosis Endothelial injury Plaque rupture Intravascular thrombosis	Radiation dose reduction Statins RAS inhibitors ^b^ Low-dose aspirin ^a^ Colchicine ^b^	[135,138,139,140,141,142,143,144,145,146,147] [4,158,159,160,161,162,163] [135,164,165] [169,170,171,172,173] [135,177,178]

^a^ selected cases; ^b^ non-human studies; 5-FU, 5-fluorouracil; VEGF, vascular endothelial growth factor; VEGFR, vascular endothelial growth factor receptor; DHP, dihydropyridines; RAS, renin-angiotensin system.

## Data Availability

Not applicable.
